# Tissue microRNAs in non-small cell lung cancer detected with a new kind of liquid bead array detection system

**DOI:** 10.1186/s12967-020-02280-5

**Published:** 2020-03-02

**Authors:** Yuan-Yuan Zheng, Yun Fei, Zheng Wang, Yue Chen, Cheng Qiu, Fu-Rong Li

**Affiliations:** 1 Department of Pathophysiology, The Basic Medical School, Jinan University, Guangzhou, China; 2 Translational Medicine Collaborative Innovation Center, The Second Clinical Medical College (Shenzhen People’s Hospital), Jinan University, No. 1017 Dongmen North Road, Shenzhen, 518020 China; 3 Department of Clinical Diagnosis Laboratory, The Second Clinical Medical College (Shenzhen People’s Hospital), Jinan University, Shenzhen, China; 4 Department of Surgery, The Second Clinical Medical College (Shenzhen People’s Hospital), Jinan University, Shenzhen, China; 5 Institute of Respiratory Diseases, The Second Clinical Medical College (Shenzhen People’s Hospital), Jinan University, Shenzhen, China

**Keywords:** miRNAs, Liquid bead array, Lung cancer, Tumor tissue

## Abstract

**Background:**

Commonly used miRNA detection methods cannot be applied for high-throughput analyses. However, this study was aimed to performed a liquid bead array detection system (LBAS) to detect tissue 6 miRNAs in non-small cell lung cancer (NSCLC).

**Methods:**

In this study, evaluation of LBAS was performed to observe the precision, specificity, limitation and stability. Then, a total of 52 primary NSCLC patients who received resection operation without preoperative radiotherapy and chemotherapy between June 2013 and March 2014 were selected, and then the total RNA of the tissues were extracted. We prepared six NSCLC-related miRNAs for LBAS. After optimization and evaluation, LBAS was verified by detecting the relative expression levels of 6 microRNAs in the pathological tissues and corresponding normal tissues of 52 NSCLC patients.

**Results:**

The results of evaluation of LBAS showed that the Mean Fluorescence Intensity (MFI) of the reaction only added with chimeric probes and beads showed no significant change after 180 days (*P *> 0.05). And the intra-assay Coefficient of Variation (CV) was between 1.57 and 3.5%, while the inter-assay CV was between 4.24 and 11.27%, indicating this system was ideal for diagnostic reagents. In addition, only the beads corresponding to the additional miRNAs showed high MFIs from 8426 to 18,769, whereas the fluorescence values of the other beads were under background levels (MFIs = 20 to 55) in each reaction, indicating no cross reactivity among the miRNAs. The limit of detection of miR-21, miR-210, miR-125b, miR-155, miR-375, and miR-31 were 5.27, 1.39, 1.85, 2.01, 1.34, and 2.73 amol/μL, respectively, showing that the lowest detection limit of miRNA by this system was under pM level. Then, the relative expression levels of miR-21, miR-210, miR-125b, miR-155, miR-375, and miR-31 by using this system were significantly correlated with NSCLC (*P *< 0.05). And the results of AUC method indicated that specific of the LBAS system was 94.2%.

**Conclusions:**

Our findings suggest that LBAS was simple, high-throughput, and freely combined with absolute quantification. Thus, this system could be applied for tumor miRNAs detection.

## Introduction

MicroRNAs (miRNAs) are endogenous small non-coding RNA molecules; more than half of the genes regulated by miRNAs are located in tumor-associated genomic regions or fragile sites, which are crucial in the development and progression of tumor [1]. Numerous studies have shown that specific miRNAs can be detected in lung cancer tissue, peripheral blood, pleural effusion, and sputum; dozens of miRNAs have been correlated with lung cancer [2]. However, the results varied among different experimental groups and showed poor comparability. Such discrepancies were mainly ascribed to the length of miRNAs (small size) and the choices of internal reference, which limits the development of miRNA detection technology. The detection methods for miRNA commonly include Northern blot analysis, RT-qPCR, microarray, miRvial [3], ratiometric electrochemical assay [4], electrochemical DNA hydrogel biosensor [5] and a microplate-based enhanced chemiluminescence system [6]. Northern blot analysis is the gold standard, but this method is time-consuming; RT-qPCR involves a complex stem-loop primer design and cannot perform multiple detection despite its high sensitivity; microarray is a high-throughput detection method with low specificity and reproducibility, thus making it unsuitable for clinical applications [7, 8]. Many differences also exist in the design principle of chips and RT-qPCR kits, leading to relatively different results.

Liquid chip technology demonstrates some advantages, such as high-throughput screening, easy operation, non-requirement of pretreatment and amplification, and indicators are selected flexibility based on a combination of individual needs, hence significantly reducing testing costs. Dacic et al. [9] used a first-generation FlexmiR to test 319 kinds of mature miRNAs from lung adenocarcinoma and adjacent tissues; they found that the expression of miR-20a, miR-328, miR-34c, and miR-18b increased by more than 20 times in cancer tissues than in adjacent tissues, whereas miR-32, miR-137, and miR-342 reduced by more than 20 times. Markou et al. [10] applied the same method to analyze the expression profiles of non-small cell lung cancer (NSCLC) tumor and adjacent tissues; however, only miR-21 expression increased. Wang et al. [11] selectively detected miR-21, miR-31, miR-222, miR-145, and miR-126 in NSCLC tumor and adjacent tissues by using the second-generation FlexmiR and found miR-21, miR-31, and miR-222 were up-regulated, while miR-145 and miR-126 down-regulated. Validation of the qRT-PCR results showed high correlation between the findings of the two techniques, thereby confirming the reliability of FlexmiR [9–11]. However, this study is importantly based on our previous research authorized to a patent [12] [ZL201510256039.4], in which six NSCLC-related miRNAs (miR-21, miR-210, miR-125b, miR-155, miR-375, and miR-31) were statistically screened as detection indicators. In addition, these six miRNAs were selected as they were reported in numbers of researches and their expression between normal people and NSCLC patients were the most significant through statistical analysis of the results of miRNAs published in various types of NSCLC samples (including tissue, peripheral blood and sputum) since 2010. Therefore, in this research we would indicate that LBAS would be established for the early diagnosis of lung cancer and would be verified onmiRNAs in cancer tissues from 52 patients at different stages of NSCLC, in order to explain that this technology would be simple, high-throughput, and freely combined with absolute quantification.

## Materials and methods

### Patients and tumor tissues

A total of 52 primary NSCLC patients (40 adenocarcinoma, 8 squamous-cell carcinoma, and 4 large-cell lung cancer) who received resection operation without preoperative radiotherapy and chemotherapy between June 2013 and March 2014 in Department of thoracic surgery, the Second Clinical Medical College of Jinan University (Shenzhen People’s Hospital) were selected. All the cases were confirmed by pathology. The patients ranged from 38 to 83 years old (average age was 62.8 years), including 40 males and 12 females. Up to 24, 12, and 16 cases of stage I, II, and III–IV NSCLC, respectively, without distant metastases were included. All subjects gave their informed consent for inclusion before they participated in the study. The study was conducted in accordance with the Declaration of Helsinki, and the protocol was approved by the Ethics Committee of Shenzhen People’s Hospital.

The parts of the cancerous tissues where tumors grow actively without necrosis and adjacent tissues of 0.5 cm to 2 cm distance from the tumor were surgically resected. Tissues were frozen in liquid nitrogen quickly within 10 min after leaving the body and transferred to a − 70 °C refrigerator within 30 min for long-term preservation.

### Total RNA extraction

Lung squamous cancer cells (Sk-mes-1 cells) were purchased from Shanghai Bioleaf Biotechnology Company. The cells were cultured for 3 to 5 days then counted to 1 × 10^6^, and 1 mL of RNAiso Plus (Takara, Japan) was added. RNAiso Plus was added into the cancer and adjacent tissues at 50 mg/mL to 80 mg/mL concentration after grinding the tissues with liquid nitrogen into powder. Total RNA was extracted and dissolved in 20 μL of RNase-free ddH_2_O (Takara, Japan) in accordance with the kit instructions. RNA quantification was assessed using BioSpectrometer (SpectroArt200S, WEALTEC, Nevada, USA), and the RNA was immediately kept in − 80 °C ultra-low temperature freezer.

### Experimental procedures

Dysregulated miRNAs in NSCLS tissue have been reported by more than 1 study [13–18]. miR-21 [13], miR-210 [14], miR-375 [15], miR-125b [16], miR-155 [17], and miR-31 [18] were differentially expressed in NSCLC tissues; combinations of these markers were set as detection indicators.

Six MagPlex^®^-TAG™ microspheres (Luminex, Austin, USA) were randomly selected, and six DNA-RNA chimeric probes (Takara, Japan) were designed on the basis of sequences of the previously selected the six miRNAs (miRBase) and anti-TAG on MagPlex^®^-TAG™ microspheres. The chimeric probes comprised RNA sequences that are 100% complementary to target miRNAs and a DNA sequence, which is 100% complementary to specific anti-TAG sequences; all these sequences should be biotinylated at the RNA 5’ end (Table 1).

**Table 1:** Chimeric probes for NSCLC relative-miRNAs detected by Luminex 200

miRNA	Mature miRNA sequence	Chimeric probe sequence	Bead number
miR-21	5′-UAGCUUAUCAGACUGAUGUUGA-3′	5′Biotin-UCAACAUCAGUCUGAUAAGCUATACATTCAACACTCTTAAATCAAA-3′	Region26
miR-210	5′-CUGUGCGUGUGACAGCGGCUGA-3′	5′Biotin-UCAGCCGCUGUCACACGCACAGCACTTAATTCATTCTAAATCTATC-3′	Region28
miR-125b	5′-UCCCUGAGACCCUAACUUGUGA-3′	5′Biotin-UCACAAGUUAGGGUCUCAGGGACACTACACATTTATCATAACAAAT-3′	Region42
miR-155	5′-UUAAUGCUAAUCGUGAUAGGGGU-3′	5′Biotin-ACCCCUAUCACGAUUAGCAUUAAAATCAACACACAATAACATTCATA-3′	Region48
miR-375	5′-UUUGUUCGUUCGGCUCGCGUGA-3′	5′Biotin-UCACGCGAGCCGAACGAACAAATTAATACAATTCTCTCTTTCTCTA-3′	Region54
miR-31	5′-AGGCAAGAUGCUGGCAUAGCU-3′	5′Biotin-AGCUAUGCCAGCAUCUUGCCUCTAAACATACAAATACACATTTCA-3′	Region62

*NSCLC* non-small cell lung cancer

Total RNA was diluted to 800 ng/μL for inspection. The final concentration of each probe in the mixed probe working dilution, which should be prepared freshly for each experiment, was 10 fmol/μL. 1.5 × TMAC was used for hybridization, containing 4.5 M TMAC, 0.15% Sarkosyl solution, 75 mM Tris–HCL, and 6 mM EDTA. Approximately 2.5 μL of the sample (Total RNA or H_2_O for negative control), 16.25 μL of 1.5 × TMAC, and 1.25 μL of the chimeric probes’ mixed dilution were pipetted to appropriate wells of a 96-well PCR plate sealed by MicroSeal A (Bio-Rad, California, USA); the plate was vortexed for 5 s, followed by a quick spin to ensure that all reagents settled at the bottom of the wells. The 96-well PCR plate was placed in a thermal cycler programmer (Bio-Rad, California, USA) with the step-down protocol as follows: 90 °C for 3 min, 80 °C decreased by 1 °C every 6 min until 60 °C was achieved, and 37 °C on hold. About 4 µl of the bead mix (2500 beads/region) was added to each well at 37 °C hold step. The wells were vortexed for 10 s to 15 s, quickly spun for 1 s to 2 s, and incubated at 37 °C for 30 min. The wells were mixed by gentle vortexing after the addition of 2.5 µL of 1:500 dilution of RNase A (Sigma, USA) to each reaction, followed by incubation at 30 °C for 30 min. The plate was placed on a magnetic separator (Luminex, Austin, USA) to remove the reaction supernatant prior to the addition of 75 µL of SAPE (Invitrogen, USA) working solution (2 ng/μL). Subsequently, the plate was shaken for 30 min at room temperature. The plate was washed again, and 100 µL of 1.5 × TMAC was added to resuspend the beads. The mean fluorescence intensity (MFI) was read by Luminex 200 (Luminex, Austin, USA). Two blanks were set up for each experiment, and each sample was repeated thrice.

### Standard curve establishment

The miRNA mimic (Takara, Japan) mixture was fold-diluted at 10 pmol/μL, 1 pmol/μL, 100 fmol/μL, 10 fmol/μL, 1 fmol/μL, 100 amol/μL, 10 amol/μL, and 1 amol/μL. Six consecutive concentrations were selected for each test as previously described by using Logistic 5P regression model to draw the standard curve in xPONENT 3.1 software. The optimal standard curve was determined by the determination coefficient *R*^2^. A larger *R*^2^ resulted in better goodness of fit of the curve. After determining the optimal standard curve, xPONENT 3.1 software automatically converted MFIs for the corresponding miRNA concentrations.

### Performance evaluation of miRNA Luminex detection system

Six target miRNAs of 1 × 10^6^ SK-MES-1 cells were detected simultaneously for seven times to calculate the intra-assay coefficient of variation (CV). These target miRNAs were tested continuously for 7 days to calculate the inter-assay CV. The six kinds of MagPlex^®^-TAG™ microspheres and six types of chimeric probes were hybridized with each miRNA mimic separately to verify the specificity of the reaction. Mean and standard deviations of the MFIs of the six miRNAs in 20 blanks were counted, pluggings to a standard curve equation to calculate the concentration, which is the limit of detection (LOD) respectively. The miR-21 mimics, corresponding chimeric probes and region 26 beads, were stored for 6 months at − 70 °C and 4 °C, respectively. The MFIs of 10 fmol/μL chimeric probes hybridizing with beads, 100 fmol/μL miRNA mimics, and 10 fmol/μL chimeric probes hybridizing with beads were detected following the above methods at 0, 30, 90, and 180 days. The probes and mimics were degraded depending on the changes in fluorescence signal. Each experiment was repeated thrice.

### miRNA detection in tissues by Luminex

miR-21, miR-210, miR-125b, miR-155, miR-375, and miR-31 expression of NSCLC and adjacent tissues was detected following the method described above. Each experiment was repeated thrice.

### Statistical analysis

IBM SPSS Statistics 25 software was used for statistical analysis of all the data. Normality of data were analyzed and homogeneity of variances were detected using exploratory analysis in descriptive statistics. If the data did not conform to the normal distribution and the variances were different, detection results were expressed using Mann–Whitney U-Test to determine the significant differences of values between groups, otherwise, detection results were expressed using Student’s t test. For paired-samples, relationships between paired-samples were tested and Wilcoxon signed ranks test was used to detect the significant differences of values between paired-samples. Pearson’s correlation analysis was applied to assess the relationship between miRNA concentration and demographic characteristics of the patients. Receiver-operating characteristic (ROC) curve and area under the curve (AUC) analyses were performed to determine the accuracy of each miRNA in the specimens and Precision-recall (PR) curve were also performed to add additional evaluation of predictive value of the approach. Statistical significance was set at P < 0.05.

## Results

### Standard curve

**Fig. 1:**
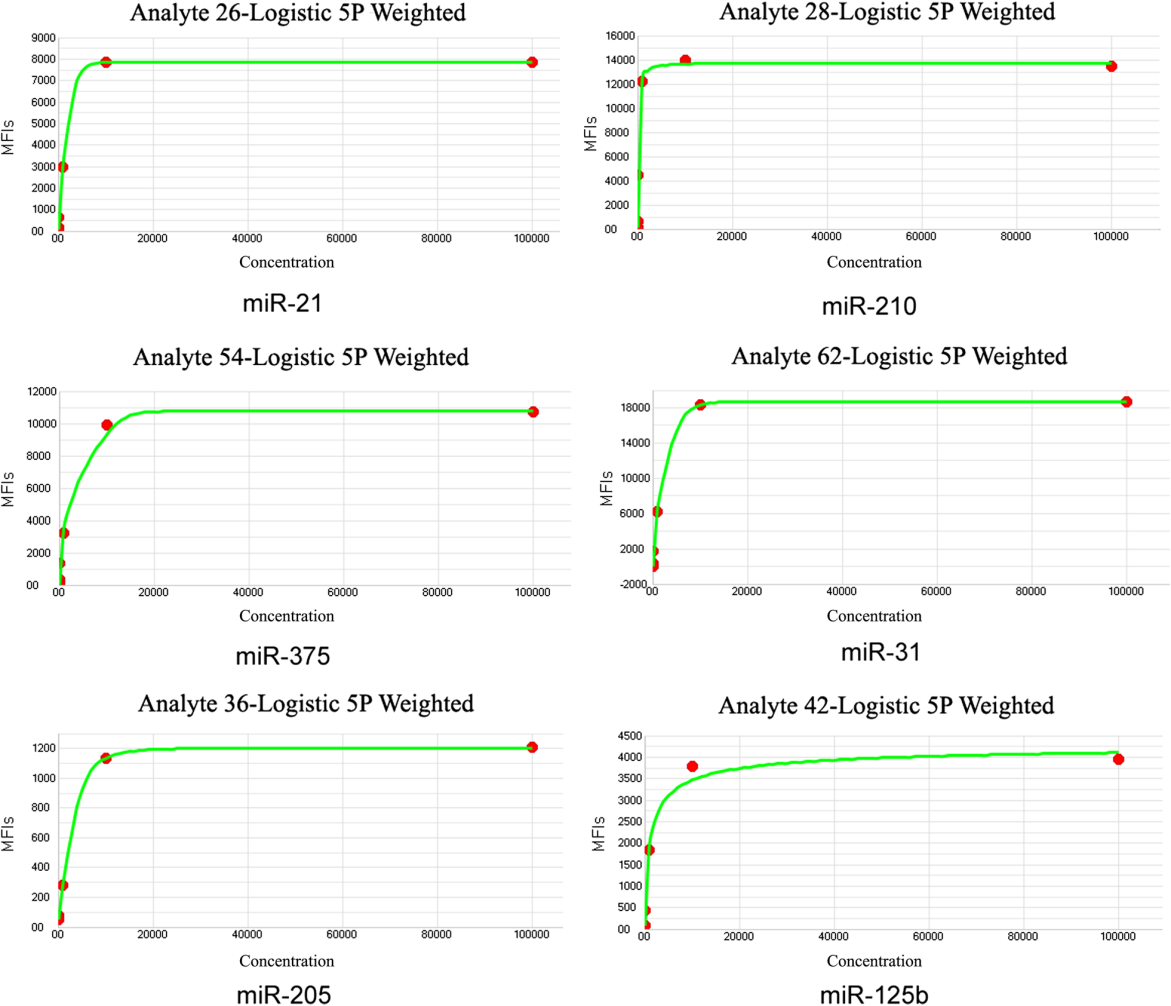
Standard curves of six miRNAs. Standard curve was drawn by xPONENT 3.1 using miRNA mimics, the concentration of which ranged from 100 fmol/μL to 1 amol/μL. The ordinate is the concentration of each miRNA mimic, and the abscissa is the mean fluorescence intensity detected by Luminex 200

Standard curve was drawn by six concentrations, namely, 100 fmol/μL, 10 fmol/μL, 1 fmol/μL, 100 amol/μL, 10 amol/μL, and 1 amol/μL, when the probe concentration was 10 fmol/μL and *R*^2^ > 0.99, thereby confirming the goodness of fit of the curve (Fig. 1).

### Performance evaluation of miRNA Luminex detection system

Precision: The intra-assay CV was between 1.57 and 3.5%, and the inter-assay CV was between 4.24 and 11.27%. These findings conform to the intra-assay CV < 5% and inter-assay CV < 10%, which are ideal for diagnostic reagents (Table 2).

**Table 2:** Intra-assay and inter-assay CV (%) of miRNAs detected by Luminex 200

miRNAs	Inside CV (%)	Between group CV (%)
miR-21	2.03	6.33
miR-210	1.72	4.24
miR-125b	2.11	11.27
miR-155	3.39	8.96
miR-375	2.94	7.04
miR-31	1.57	5.85

*CV* coefficient of variation

Specificity: Only the beads corresponding to the additional miRNAs showed high MFIs from 8426 to 18,769, whereas the fluorescence values of the other beads were under background levels (MFIs = 20 to 55) in each reaction. These findings indicated no cross reactivity among the miRNAs (Table 3).

**Table 3:** Fluorescence signal of the reaction between 6 microspheres and different miRNAs

miRNA standards	Region26	Region28	Region36	Region42	Region48	Region54	Region62
miR-21	18,769	53	51	44	42	36	49
miR-210	21	15,942	26	55	50	46	27
miR-125b	47	41	35	10,988	49	55	23
miR-155	20	45	26	27	17,743	42	33
miR-375	32	47	40	39	38	16,458	45
miR-31	22	19	38	36	46	52	18,527

Region, the six kinds of MagPlex^®^-TAG™ microspheres

Limit of detection: The limit of detection of miR-21, miR-210, miR-125b, miR-155, miR-375, and miR-31 were 5.27, 1.39, 1.85, 2.01, 1.34, and 2.73 amol/μL, respectively. These findings showed that the lowest detection limit of miRNA by Luminex was under pM level.

Stability: As the time went for 0 month, 1 month, 3 months and 6 months, the MFI of the reaction only added with chimeric probes and beads were separately (23,269 ± 263), (20,405 ± 191), (20,037 ± 224) and (21,884 ± 284), showing no significant change after 180 days (*P *> 0.05) (Table 4) and indicating that the probes will not degrade at − 70 °C in 6 months. By contrast, the MFI which were separately (18,227 ± 201) for 0 month,(17,475 ± 193) for 1 month,(10,937 ± 182) for 3 months and (7295 ± 134) for 6 months decreased significantly after 90 days when the miRNA mimics and RNase were added into the reaction solution (*P *< 0.05) (Table 5), indicating that miRNA mimics will gradually degrade in about 3 months.

**Table 4:** The fluorescence signal change of hybridization between microspheres and chimeric probes with time

Time (month)	MFI
0	23,269 ± 263
1	20,405 ± 191
3	20,037 ± 224
6	21,884 ± 284

**Table 5:** The fluorescence signal change of hybridization among miRNA standards, microspheres and chimeric probes with time

Time (month)	MFI
0	18,227 ± 201
1	17,475 ± 193
3	10,937 ± 182
6	7295 ± 134

### NSCLC specificity of miRNA verification

**Fig. 2:**
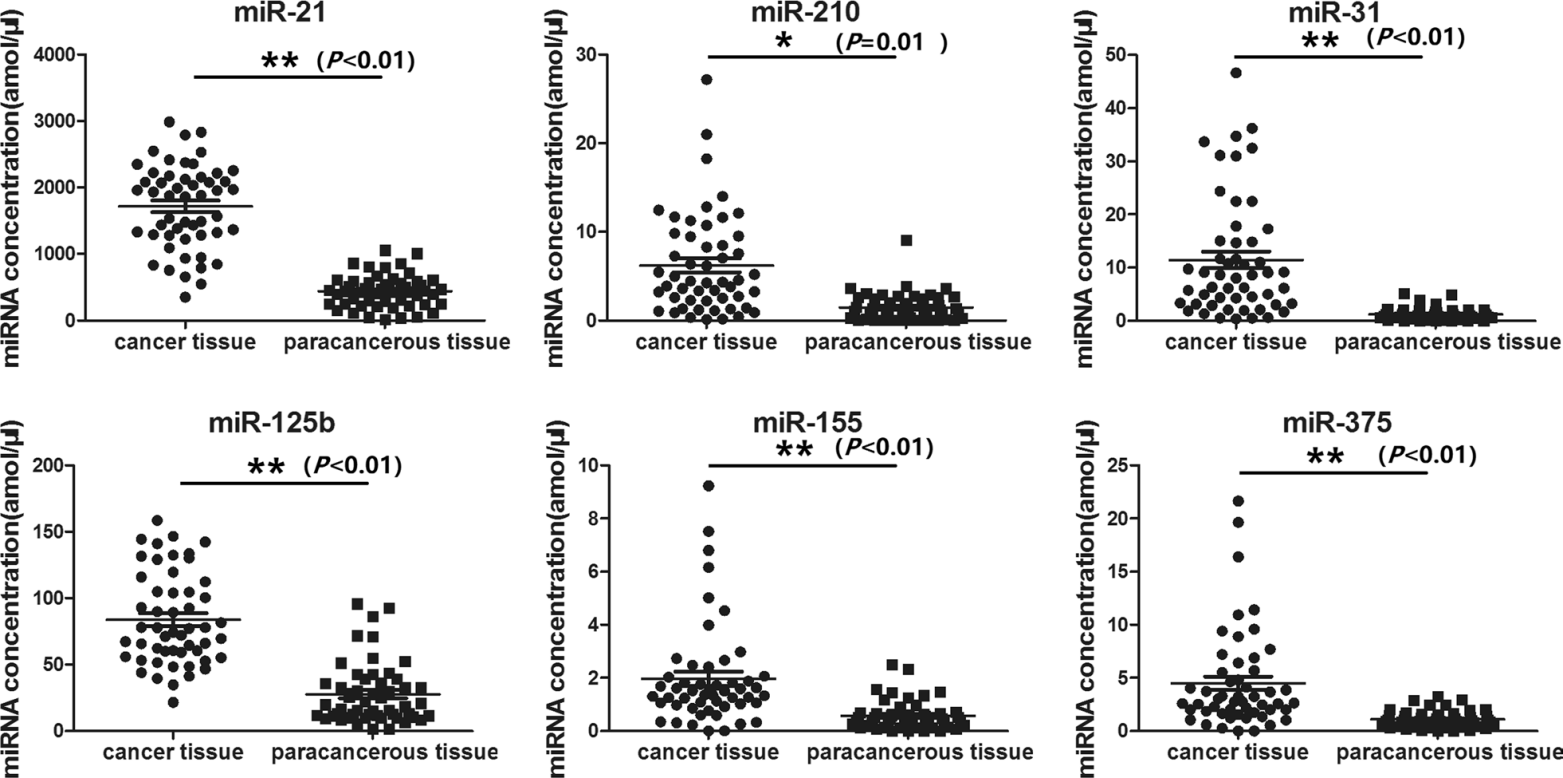
Expression level of tissue miRNAs. Expression levels of miRNAs were assessed from cancer tissues and paracancerous tissues of 52 NSCLC patients using Luminex (amol/μL). Compared with paracancerous tissues, the average expressions of miR-21, miR-125b, miR-155, miR-375, and miR-31 showed significant increase in NSCLC tissues (*P *< 0.01), and the average expression of miR-210 showed obvious increase in NSCLC tissues (*P* < 0.05)

Comparison of the average expressions of the six miRNAs in NSCLC tissues and paired adjacent tissues showed that the average expressions of miR-21, miR-210, miR-125b, miR-155, miR-375, and miR-31 were high correlated between NSCLC tissues and paired adjacent tissues (*P *< 0.01; the correlation coefficients were separately 0.71, 0.698, 0.487, 0.375, 0.399 and 0.453). Normality test was also performed and the results of Shapiro–wilk test (N > 50) showed that all the *P* value of the groups were less than 0.05, indicating that the samples did not obey normal distribution. Homogeneity test of variance was performed and the results showed all the *P* value of the groups were less than 0.05, indicating that the variances were different. Further difference analysis showed that these six miRNAs exhibited high expression in NSCLC tissues, in which the average expression of miR-210 was obviously different (*P *< 0.05) between the adjacent and cancer tissues. The other five miRNAs showed significant differences (*P *< 0.01) between the NSCLC tissues and adjacent tissues. These results indicated that the six miRNAs were significantly correlated with NSCLC (Fig. 2).

**Fig. 3:**
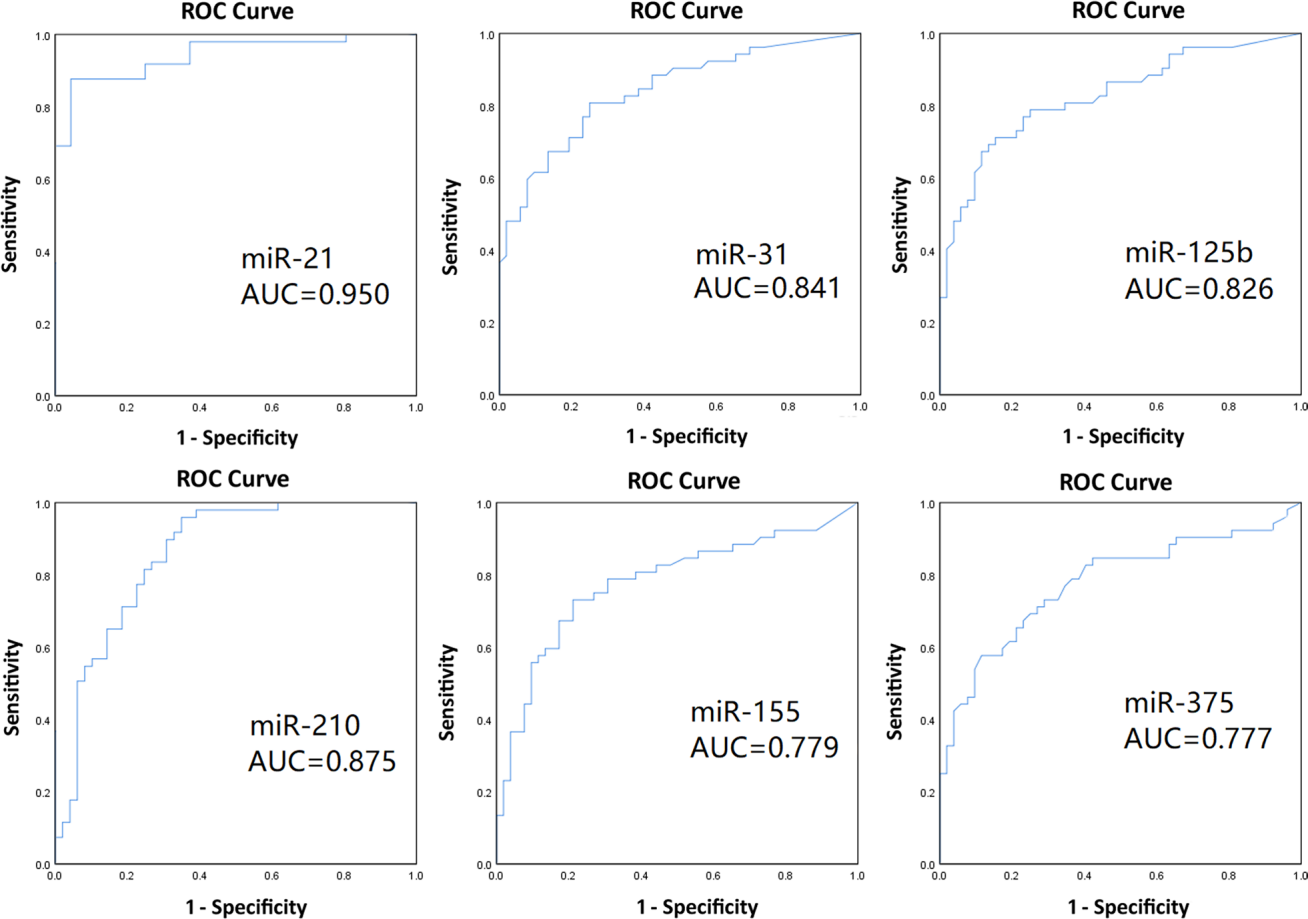
ROC curve analysis for discriminating NSCLC from controls. Receiver-operating characteristic curve analysis of expression levels of the six miRNAs (miR-21, miR-125b, miR-155, miR-375, miR-31, and miR-210) in tissues of 52 NSCLC patients. miR-21, miR-210, and miR-31 produced 0.950, 0.875 and 0.841 area under the curve (AUC) values, respectively, which are higher than AUC values from the other miRNAs

The ROC curves of the expressions of the six miRNAs in cancer tissues were measured to determine their clinical values. So the expressions of the six miRNAs in the tumor group and the adjacent group were showed by ROC curves analysis and the results showed that the first four miRNAs with optimal specificity were miR-21, miR-210, miR-125b and miR-31; the AUC values were 0.950, 0.875, 0.826 and 0.841, respectively (Fig. 3 and Table 6). According to the ROC curve, the best cut-off value is the point of which the Youden index is the largest, implying the high sensitivity and specificity of the test and the small rate of misdiagnosis and missed diagnosis. So, the best cut-off values of the six miRNAs-miR-21, miR-31, miR-210, miR-155, miR-375, and miR-125b were seperately 727.02 (sensitivity: 96.2%; specificity: 100%), 2.315 (sensitivity: 73.1%; specificity: 88.2%), 43.035 (sensitivity: 92.3%; pecificity: 41.2%), 1.05 (sensitivity: 76.9%; specificity: 76.5%), 2.455 (sensitivity: 69.2%; specificity: 76.5%) and 3.2 (sensitivity: 61.5%; specificity: 100%) (Table 6). The total predicted accuracy of the predictive model (judging the quality of diagnosis by the created LBAS system) established by miR-21, miR-210, and miR-31 was 94.2%, which was analyzed by non-conditional logistic regression with using SPSS software.

**Table 6:** The value of miRNAs abnormal expression for NSCLC diagnosis

miRNA	AUC	The best cutoff value	Sensitivity (%)	Specificity (%)
miR-21	0.950	727.02	96.2	100.0
miR-31	0.841	2.315	73.1	88.2
miR-210	0.875	43.035	92.3	41.2
miR-155	0.779	1.05	76.9	76.5
miR-375	0.777	2.455	69.2	76.5
miR-125b	0.826	3.2	61.5	100.0

**Fig. 4:**
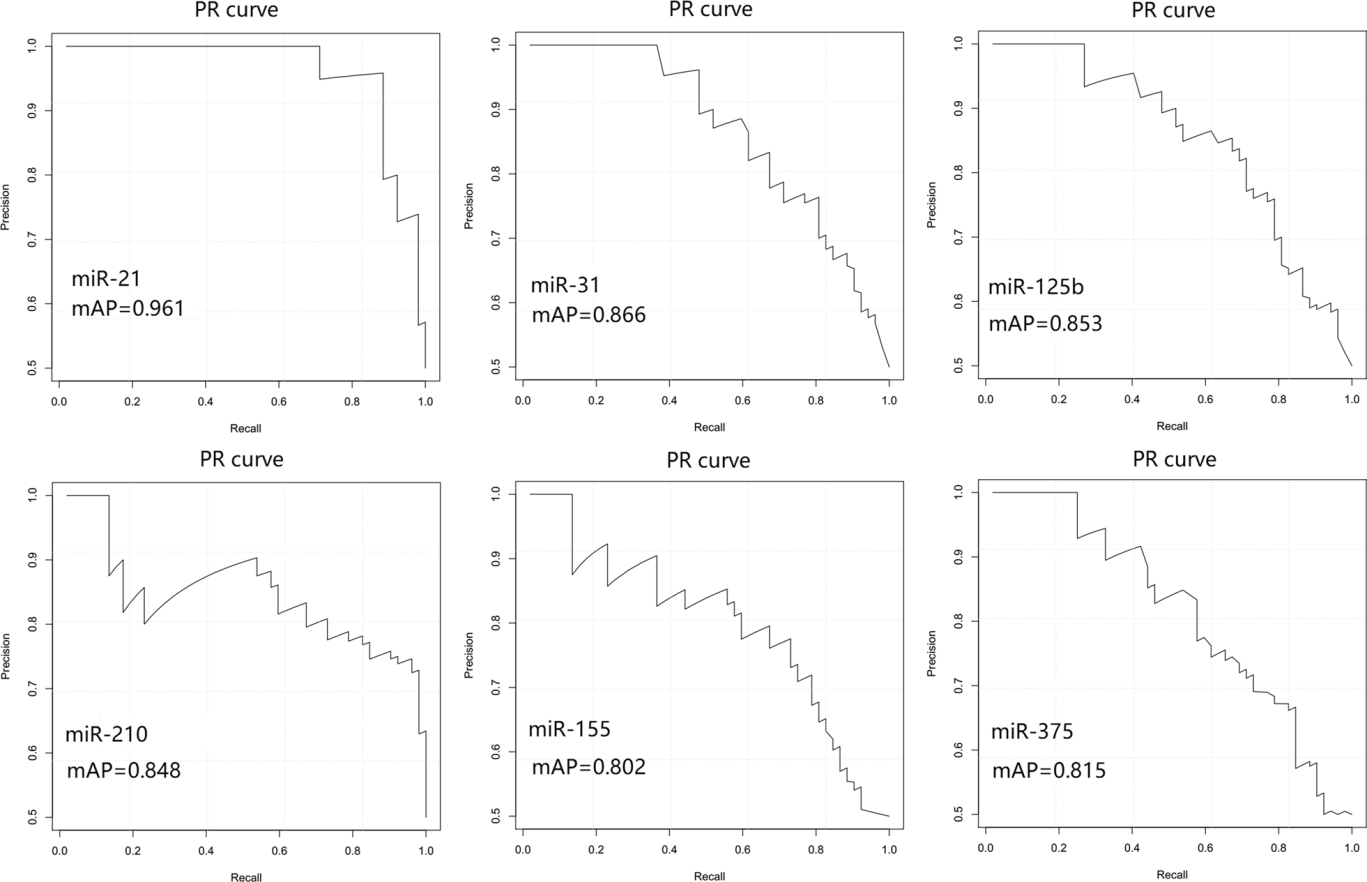
PR curve analysis for discriminating NSCLC from controls. Receiver-operating characteristic PR curve analysis of expressions of the six miRNAs (miR-21, miR-210, miR-125b, miR-155, miR-375, and miR-31) in tissues of 52 NSCLC patients

The Precision-recall (PR) curve of the expressions of the six miRNAs in cancer tissues were added to determine their clinical values by R software. The PR curve was to predict the clinical value of the approach of LBAS system. And the results showed that the mean Average Precision (mAP) of miR-21, miR-31, miR-210, miR-155, miR-375 and miR-125b were 0.961, 0.866, 0.848, 0.802, 0.815 and 0.853. The first four miRNAs with high precision were miR-21, miR-210, miR-125b and miR-31, consistent with the results of ROC curves above (Fig. 4).

### Correlation analysis of miRNA expression and clinical data

The correlation between the clinical data and the expression of the six miRNAs in tissues of 52 NSCLC patients was analyzed (Table 7). All the miRNAs showed no relationship with age and sex (*P *> 0.05); miR-21, miR-210, and miR-375 were significantly correlated with smoking history (*P *< 0.05); miR-375 was overexpressed in adenocarcinoma compared with squamous-cell carcinoma and large-cell lung cancer (*P *< 0.05); miR-21, miR-155, and miR-375 in cancer tissues with lymph node metastasis was higher than those in the non-metastasis group (*P *< 0.01). These results demonstrated an association between miRNA levels and smoking history, as well as in the different clinical stages and pathological types.

**Table 7:** Relationship between miRNAs expression and clinical parameters

Variable	No.	*P* values/correlation coefficients					
		miR-21	miR-210	miR-125b	miR-155	miR-375	miR-31
Age (years)							
≥ 60	32						
≤ 60	20	0.058/0.222	0.092/0.359	0.548/0.395	0.452/0.479	0.290/0.340	0.175/0.639
Gender							
Male	40						
Female	12	0.369/0.491	0.261/0.613	0.686/0.538	0.117/0.043	0.886/0.443	0.102/0.284
Smoking status							
Smoker	26						
Non-smoker	26	0.005/0.255	0.004/0.199	0.440/0.289	0.690/0.187	0.003/0.358	0.357/0.395
Pathological type							
AD	40						
SC	8						
LCC	4	0.172/0.183	0.352/0.114	0.804/0.462	0.825/0.055	0.028/0.157	0.062/0.704
TNM stage							
I	24						
II	12						
III–IV	16	0.098/0.298	0.212/0.238	0.296/0.151	0.117/0.117	0.067/0.300	0.728/0.320
LNM							
Yes	18						
No	34	0.006/0.305	0.205/0.413	0.056/0.926	0.000/0.181	0.002/0.423	0.693/0.072

*AD* adenocarcinoma, *SC* squamous carcinoma, *LCC* large-cell carcinoma, *LNM* lymph node metastasis

## Discussion

The miRNA detection by Luminex (FlexmiR) underwent two generations. The first-generation FlexmiR used polystyrene microspheres coupled with LNA probes designed by reverse sequence of purpose miRNA to capture the biotinylated target miRNA; fluorescent signal molecules were directly added after washing and detected in the machine. LNA and RNA hybrid duplexes were stable compared with the conventional nucleic acids. Hence, the reaction sensitivity increased. The second-generation FlexmiR was used to design the DNA-RNA chimeric probe, which consisted of miRNA and anti-TAG complementary sequences coupled with specifically encoded microspheres and formed the microsphere-chimeric probe-miRNA complexes in the reaction system. These complexes increased the reaction specificity by stepwise cooling hybridization of the RNase A characteristics, which can degrade the unbound or mismatched probes. The biotin-labeled amplification and dephosphorylation were unnecessary. Thus, the time for manual operation was reduced. As a non-traditional chip technology, Luminex offers a practical value for detecting specific miRNAs in multiple samples because of its ability for multiplex analysis and flexible selection of indicators. Compared with conventional chips, the shorter manual operation time and higher specificity yields more clinical value. Using the first-generation FlexmiR, Dacic et al. and Markou et al. analyzed the miRNA expressions of NSCLC cancer tissues and adjacent tissues and verified the results by qRT-PCR. They found that the test results of the two techniques were highly correlated, thereby confirming the reliability of FlexmiR [9–11].

On the basis of the statistical analysis results of our previous literature described in Introduction part, the current research selected miR-21, miR-210, miR-125b, miR-155, miR-375, and miR-31 as candidate makers to establish a miRNA liquid chip system. No cross reactivity existed among the miRNAs, basically in this system, in which the limit of detection was between 1.02 and 5.27 pM, and the sensitivity and specificity were basically in the ideal range. Intra-assay CV ranged from 1.57 to 3.50%, and inter-assay CV was between 4.24 and 11.27%. The inter-assay CV was mainly caused by the differences in the miRNA extraction process. First, the number of cells in each batch for the extraction of miRNAs cannot be exactly the same. Second, the extraction yield of the miRNA in each batch was not entirely consistent. Moreover, miRNA mimics were insufficiently stable and degraded within 3 months. The MFI was between 16,000 and 23,000 when the 12.5 fmol biotin-labeled probe was hybridized with 2500 microspheres only; when adding at least 10 times miRNA mimics of probe, the MFI should be about 16,000 to 23,000 because all the probes should be double-stranded combined with miRNA mimics, which cannot be resected by RNase theoretically. However, the MFI was between 7000 and 17,000 after enzyme digestion, which proved that the portion of the probes were not bound to the miRNAs but were still single-stranded and digested by RNase. The results also suggest that not all target miRNAs were captured by beads. Thus, the fluorescent signal cannot be detected when target miRNAs were micro-scaled in samples. Even if the reaction area was increased in the liquid phase, the target miRNAs cannot be bound to the probes at all because of the short miRNA sequence and large steric hindrance [19]. We can increase the sample size or pre-amplified samples to address this problem, but such increase will also raise the detection cost, extend the operating time, and lose the advantages of the liquid chip.

The established system was used to detect the following six miRNAs in NSCLC and adjacent tissues: miR-21, miR-210, miR-125b, miR-155, miR-375, and miR-31, which showed high expression in NSCLC tissues; miR-210 was obviously different between the adjacent and cancer tissues (*P *< 0.05), whereas the five other miRNAs showed significant differences (*P *< 0.01). Then, the ROC curves and PR curves of the expressions of the six miRNAs in cancer tissues were measured to determine their clinical values. The results showed that miR-21, miR-31, miR-210 and miR-125b were the most NSCLC-specific (AUC = 0.950, 0.841, 0.875, 0.826, respectively). According to the ROC curves, the best cut-off values of the six miRNAs-miR-21, miR-31, miR-210, miR-155, miR-375, and miR-125b were seperately 727.02, 2.315, 43.035, 1.05, 2.455 and 3.2. The created prediction models which were analyzed with SPSS software yielded a total accuracy rate of 94.2%. According to PR curves, the mean Average Precision (mAP) of miR-21, miR-31, miR-210, miR-155, miR-375 and miR-125b were 0.961, 0.866, 0.848, 0.802, 0.815 and 0.853. The first four miRNAs with high precision were miR-21, miR-210, miR-125b and miR-31, consistent with the results of ROC curves above. According to previous research, miR-21 and miR-210 were reported most frequently in overexpressed miRNAs of lung cancer tissues [8, 9]. miR-21 showed high expression in various tumor tissues and peripheral blood, including NSCLC, gastric cancer, and colorectal cancer [20, 21]. Over-expressed miR-21 can inhibit apoptosis by down-regulating negative regulators, such as programmed cell death 4 (PDCD4), phosphatase and tensin homolog deleted on chromosome ten (PTEN), and activate epidermal growth factor receptor (EGFR) signaling pathway closely related to NSCLC [22–24]. miR-210 was also upregulated in various solid tumor tissues, and its expression in tumor tissues and in serum and sputum showed significant diagnostic value for NSCLC [25]. And its expression was controlled by hypoxia, as regulated by hypoxia-inducible factor (hypoxia-inducible factor-1 alpha, HIF-1α); HIF-1α can directly promote the rapid growth of tumors, and over-expressed miR-210 played an important role in angiogenesis [26, 27]. This study found no correlation between miR-21 and miR-210 expression in NSCLC tissues in terms of histological types and clinical stages (*P *> 0.05). However, miR-21 expression level was higher in NSCLC with lymph node metastasis than in that without metastasis (*P *< 0.01), and miR-21 and miR-210 expression levels were higher in smoking cancer tissues than in those of non-smokers (*P *< 0.01). These findings showed that smoking maybe cause increased expression of miR-21 and miR-210, and a higher miR-21 level will result in lower survival rate; Over-expressed miR-21 also indicated poor prognosis [28]. miR-31 was specific in malignant tumor cells and characterized by strict expression pattern among tumors with different tissue origins. miR-31 decreased in breast and gastric cancers but increased in colon cancer, as well as in head and neck tumors [29–31]. In lung cancer, miR-31 played a role in tumorigenesis by inhibiting large tumor suppressor 2 and other tumor suppressor genes [32]. miR-31 expression was significantly higher in NSCLC tissues (*P *< 0.01) but unrelated to pathological types, clinical stages, lymph node metastasis, and smoking history (*P *> 0.05). miR-125b, a human homologue of lin-4, was reported to be involved in regulating tumor cellular proliferation and tumor progression [33–37]. In NSCLC, the increased miR-125b could be positively associated with NSCLC stages and poor patient survival [9, 10]. In addition, our research observed that miR-125b expression in NSCLC tumor tissues was significantly higher than those in adjacent tissues. These miRNAs, namely, miR-21, miR-210, miR-125b and miR-31, were closely correlated with the occurrence and development of NSCLC. These miRNAs also exhibit potential application values in the early diagnosis of lung cancer.

Blood and body fluids were ideal for disease screening,as previous studies have shown that despite the large amount of RNase in the blood and sputum, miRNAs can be stable to pH, temperature, repeated freezing, and strong thawing. Among them, miR-21, miR-210, miR-125b and miR-31 were not only significantly increased in various types of early lung cancer, but also differentially expressed in the peripheral blood and sputum of lung cancer patients [38–42]. However, whether miR-21, miR-210, miR-125b and miR-31 in sputum or blood can be used as early screening markers for NSCLC would be fully verified in a later large-sample, multi-center clinical study. Consequently, further research for technical optimization and sensitivity, specificity, stability, as well as a large-sample clinical validation study on miRNAs in serum or sputum, is necessary to apply the NSCLC-specific miRNA Luminex detection system for early screening of NSCLC.

## Conclusion

In conclusion, LBAS showed favorable standard curve, and improved reproducibility and specificity; miRNAs in cancer tissues from 52 patients at different stages of NSCLC were detected by LBAS, indicating that miRNA was up-regulated in NSCLC cancer tissues (*P *< 0.05); the accuracy of the joint detection was 94.2%. These results showed that LBAS could be applied for tumor miRNAs detection. And this technology was not only stable and special but also simple, high-throughput, and freely combined with absolute quantification.

## Data Availability

The datasets used and/or analysed during the current study are available from the corresponding author on reasonable request.

## Abbreviations

LBAS: Liquid bead assay detecting system
LC: Lung cancer
miRNAs: microRNAs
NSCLC: Non-small cell lung cancer
CV: Coefficient of variation
LOD: Limit of detection
MFI: The mean fluorescence intensity
ROC: Receiver-operating characteristic curve
AUC: Area under the curve
PDCD4: Programmed cell death 4
PTEN: Phosphatase and tensin homolog deleted on chromosome ten
EGFR: Epidermal growth factor receptor
HIF-1α: Hypoxia-inducible factor-1 alpha
